# My Life (Poetry)

**Published:** 1882-01

**Authors:** Francis Gerry Fairfield


[From the Home Journal.]

### MY LIFE.

BY FRANCIS GERRY FAIRFIELD.

My soul is like a lute that moans
In low, mysterious undertones;
I listen, try to comprehend
The notes that murmur, mix and blend.

"Ah, life," unto myself I say,
"A dream by night, a dream by day;
And just as actual doth seem
The sleeping as the waking dream.
An image in a stagnant pool
I see and call it beautiful;
A journey every man must wend,
With nothingness at either end.
Ripe grapes, that when I would express
Their juices, turn to nothingness;
An object lens so badly ground,
No focal distance can be found.
A voyager who, gliding down
A river, stops at many a town;
And finds them cities of the dead,
Only by tombstones tenanted.
A boatman who as he doth row
Doth see another boat below;
A phantom man sits at the oar,
And mocks his movements evermore.
A strolling organist, who plays
The same old tune for days and day;
A block of Parian statue-stone,
Suggesting something left unhewn.
A pert poll-parrot's ha-ha, half
Suggestive of a seraph's laugh;
A sweet harmonic tissue blent
With broken notes of discontent.
A stanza wove of different feet,
A germ within a grain of wheat;
A shell that ever must repeat
The deep, strange music of the main
Through ocean caves where it hath lain.

"He heareth best who listens not,
He seeth best who never sought
To see the things that folded lie
Between the eyelid and the eye.

"Ah, soul, a spectre that I see
Within myself, which mocks at me;
A shadow that life throws upon
The wall a moment, and is gone.

"I sit and dream. A tombstone white
Gleams in the graveyard through the night;
And they who read the epitaph
Repeat it with a smothered laugh.

"I hear no footsteps on the grass,
As heavy-hearted mourners pass;
Sad sorrowers who, by and by,
Will sleep as dreamlessly as I."

Thus spoke I to my soul. It said,
"In me man riseth from the dead;
I am the statue yet unhewn
That lies within the block of stone"

COMETS' TAILS.—Professor Ennis, of the Naval Observatory at Washington, believes that the tails of comets are electric light. "If these tails had any substance," he argues, "the laws of motion are constantly violated by them. The great comet of 1843 went so near the sun that it passed from one side to the other in a few hours. Its immense tail, 100,000,000 miles long, was shifted completely, so that it pointed directly in an opposite direction. Could that be so if it were composed of any substance? Could a comet swing 100,000,000 miles of tail around so quick as that? The electricity is generated by evaporation. As the comets approach the sun, the heat becomes more intense, the evaporation and accumulation of electricity more rapid, the repulsive force greater and the tails longer. Sometimes the material becomes completely evaporated. Then the comet has no tail."

### AT YORKTOWN.

The Yorktown celebration recalls the memory of old "Uncle Nelse," a colored veteran who used to act as guide over the field at Yorktown "befo' de wah." He had heard the story of the surrender so often, and talked it over so much, that early in life he became persuaded that he was really there and saw the whole affair, and a dialogue with a party of visitors would run in this wise:

"So, you're real certain, uncle, that you were here and saw it all?"

"O, sartin fo' shuah, massa."

"Is it possible! Well, now, tell us all about it."

"Well, you see, Massa Lawd Cawnwallis he stood right dah, and Massa Gineral Washington he stood right heah. Jes' so soon ez Massa Lawd Cawnwallis seen Massa Gineral Washington, he pulled of his hat and he sez, sez he:

"'Good-mawnin, Massa Gineral Washington.'

"'Who is you?' sed Massa Gineral Washington, looking very cross.

"'Why, I'se Lawd Cawnwallis, sah, sez he, a-bowin' and a-scrapin'.

"'*Is ye, ye son-of-a-gun?*' sez Massa Gineral Washington; *and pullin' out his sword he chopt his head clean off.*"—*Toledo Blade.*

---

A Western statistican has calculated that the annual loss to the country by fires set by the sparks of locomotives is between twenty and thirty millions of dollars; and yet it has been shown by the successful and prolonged experiment of the London Underground Railroad Company that locomotives can be fitted at mall expense so as to consume their own sparks and smoke without any necessary loss of speed.

From "Mary, the Little Missionary:" Mary's Uncle Charles came to see her, and gave her a bright gold dollar. Then Mary said: "I will buy some candy and some chewing-gum, and a pickled lime, and I will give Sarah Jones two cents, and the poor woman with the little baby three cents." But Mr. A. Sleek, that good man, heard her, and he groaned and said: "Mary, remember the Pottawottamies!" So she gave her dollar to good Mr. Sleek for the Pottawottamies, and when he took it he was kind enough to say that he wished the Pottawottamies might get it. And Mary was made a life member of Mr. Sleek's society. Was not that better than a pickled lime?—*Vanity Fair Juvenile Library.*

